# Genome Sequences of 19 Novel *Erwinia amylovora* Bacteriophages

**DOI:** 10.1128/genomeA.00931-17

**Published:** 2017-11-16

**Authors:** Ian N. D. Esplin, Jordan A. Berg, Ruchira Sharma, Robert C. Allen, Daniel K. Arens, Cody R. Ashcroft, Shannon R. Bairett, Nolan J. Beatty, Madeline Bickmore, Travis J. Bloomfield, T. Scott Brady, Rachel N. Bybee, John L. Carter, Minsey C. Choi, Steven Duncan, Christopher P. Fajardo, Brayden B. Foy, David A. Fuhriman, Paul D. Gibby, Savannah E. Grossarth, Kala Harbaugh, Natalie Harris, Jared A. Hilton, Emily Hurst, Jonathan R. Hyde, Kayleigh Ingersoll, Caitlin M. Jacobson, Brady D. James, Todd M. Jarvis, Daniella Jaen-Anieves, Garrett L. Jensen, Bradley K. Knabe, Jared L. Kruger, Bryan D. Merrill, Jenny A. Pape, Ashley M. Payne Anderson, David E. Payne, Malia D. Peck, Samuel V. Pollock, Micah J. Putnam, Ethan K. Ransom, Devin B. Ririe, David M. Robinson, Spencer L. Rogers, Kerri A. Russell, Jonathan E. Schoenhals, Christopher A. Shurtleff, Austin R. Simister, Hunter G. Smith, Michael B. Stephenson, Lyndsay A. Staley, Jason M. Stettler, Mallorie L. Stratton, Olivia B. Tateoka, P. J. Tatlow, Alexander S. Taylor, Suzanne E. Thompson, Michelle H. Townsend, Trever L. Thurgood, Brittian K. Usher, Kiara V. Whitley, Andrew T. Ward, Megan E. H. Ward, Charles J. Webb, Trevor M. Wienclaw, Taryn L. Williamson, Michael J. Wells, Cole K. Wright, Donald P. Breakwell, Sandra Hope, Julianne H. Grose

**Affiliations:** Department of Microbiology and Molecular Biology, Brigham Young University, Provo, Utah, USA

## Abstract

*Erwinia amylovora* is the causal agent of fire blight, a devastating disease affecting some plants of the *Rosaceae* family. We isolated bacteriophages from samples collected from infected apple and pear trees along the Wasatch Front in Utah. We announce 19 high-quality complete genome sequences of *E. amylovora* bacteriophages.

## GENOME ANNOUNCEMENT

*Erwinia amylovora* is a Gram-negative facultative anaerobic rod-shaped bacterium and the causative agent of fire blight (1), a disease that affects some members of the plant family *Rosaceae* and causes the infected areas of the plant to appear burnt (2, 3). *E. amylovora* is a member of the *Enterobacteriaceae* family, which includes many well-characterized pathogenic bacteria such as *Salmonella enterica* and *Escherichia coli*. Thus, understanding the evolution of this plant pathogen and the bacteriophages that infect it may provide insight into the evolution of the* Enterobacteriaceae* family, including other pathogenic strains.

Herein, we announce the genome sequences of 19 novel *E. amylovora* bacteriophages, vB_EamP_Frozen, vB_EamP_Gutmeister, vB_EamP_Rexella, vB_EamM_Deimos-Minion, vB_EamM_RAY, vB_EamM_Simmy50, vB_EamM_Special G, vB_EamM_Caitlin, vB_EamM_ChrisDB, vB_EamM_EarlPhillipIV, vB_EamM_Huxley, vB_EamM_Kwan, vB_EamM_Machina, vB_EamM_Parshik, vB_EamM_Phobos, vB_EamM_Stratton, vB_EamM_Joad, vB_EamM_RisingSun, and vB_EamM_Yoloswag. Samples were collected from apple and pear trees bearing symptoms of fire blight infection that were found along the Wasatch Front of Utah. Phages were amplified via enrichment culture of these samples, and resulting phages were then plaque purified by a minimum of three passages. All phages reported in this announcement infect the *Erwinia amylovora* ATCC 29780 strain.

Genomic DNA was extracted using the Phage DNA isolation kit (Norgen Biotek Corporation) and sequenced using 454 pyrosequencing (454 Life Sciences, Roche Diagnostics) or Illumina HiSeq 2500 sequencing (Illumina, 250-bp reads). Contigs were assembled using Newbler version 2.9 (Roche Diagnostics, Branford, CT) and Consed (4) for 454 pyrosequencing reads or Geneious version R8 (5) for Illumina reads. Assembled genomes were annotated using DNA Master (6) and other programs as described previously (7, 8).

The 19 phages fell into five distinct clusters according to genomic analysis. The first group included the jumbo myoviruses vB_EamM_Deimos-Minion, vB_EamM_RAY, vB_EamM_Simmy50, and vB_EamM_Special G, which share a minimum of 97.2% average nucleotide identity to one another. The second group included two jumbo myoviruses, vB_EamM_RisingSun and vB_EamM_Joad, which differ by only two putative gene products. The third group included diverse jumbo myoviruses vB_EamM_Caitlin, vB_EamM_ChrisDB, vB_EamM_EarlPhillipIV, vB_EamM_Huxley, vB_EamM_Kwan, vB_EamM_Machina, vB_EamM_Parshik, vB_EamM_Phobos, and vB_EamM_Stratton, which share a minimum of 50.5% average nucleotide identity. An additional jumbo myovirus, vB_EamM_Yoloswag, did not have any close phage relatives. Podovirus phages vB_EamP_Frozen, vB_EamP_Gutmeister, and vB_EamP_Rexella share at least 97.2% average nucleotide identity. The four jumbo myovirus groups package DNA by headful packaging based on homology of their putative terminase genes to the phiKZ terminase (9). Three of these genomically permuted myovirus groups were assigned their base pair (bp) 1 by alignment to previously published genomes by use of BLASTN (10) and Gepard (11) (Ea35-70 for the Deimos-Minion group [12], EL [13, 14] for the RisingSun group, and SPN3US [15] for the Caitlin group). vB_EamM_Yoloswag shared very little DNA homology with any other phage; therefore, its bp 1 was assigned to position its putative terminase at the beginning of the genome. The podovirus group genomes were assigned bp 1 by their relation to N4, in terms of both terminase similarity and whole-genome alignment, suggesting they have small terminal repeats.

### Accession number(s).

GenBank accession numbers for the 19 *Erwinia* bacteriophages are listed in Table 1.

**TABLE 1:** Properties of 19 novel *Erwinia amylovora* bacteriophage genomes

Phage name	GenBank accession no.	Sequencing type	Minimum–maximum fold coverage (avg read depth)	Genome length (bp)	No. of ORFs^a^	No. of tRNAs^b^	G+C content (%)
vB_EamP_Gutmeister	KX098391	Illumina	423–2,415 (662)	71,173	84	8	46.9
vB_EamP_Frozen	KX098389	454	79–1,779 (862)	75,147	92	8	46.9
vB_EamP_Rexella	KX098390	454	69–1,780 (885)	75,448	92	7	46.9
vB_EamM_Deimos-Minion	KU886225	454	61–1,780 (873)	273,501	326	NA	49.9
vB_EamM_RAY	KU886224	Illumina	335–910 (677)	271,182	319	1	49.9
vB_EamM_Special G	KU886222	454	19–1,779 (874)	273,224	324	NA	49.8
vB_EamM_Simmy50	KU886223	Illumina	150–831 (282)	271,088	322	1	49.9
vB_EamM_Caitlin	KX397365	Illumina	84–249 (174)	241,147	271	7	52.2
vB_EamM_ChrisDB	KX397366	454	66–1,780 (874)	244,840	277	11	49.4
vB_EamM_EarlPhillipIV	KX397367	Illumina	75–243 (164)	223,935	241	NA	50.6
vB_EamM_Huxley	KX397368	454	75–1,779 (880)	240,761	271	9	51.1
vB_EamM_Kwan	KX397369	Illumina	192–554 (362)	246,390	285	8	52.1
vB_EamM_Machina	KX397370	454	65–1,780 (879)	241,654	272	9	51.0
vB_EamM_Parshik	KX397371	454	64–1,779 (880)	241,050	271	10	51.0
vB_EamM_Phobos	KX397372	454	59–1,779 (873)	229,501	247	NA	49.1
vB_EamM_Stratton	KX397373	454	64–1,779 (874)	243,953	276	12	51.3
vB_EamM_Yoloswag	KY448244	Illumina	5–265 (99.5)	259,700	334	NA	46.91
vB_EamM_RisingSun	MF459646	Illumina	50–293 (138.6)	235,108	243	NA	48.32
vB_EamM_Joad	MF459647	Illumina	232–1,065 (522.2)	235,374	245	NA	48.29

a ORFs, open reading frames.

b NA, no tRNAS were identified.
